# Uncovering the miRNA-mediated regulatory network involved in Ma bamboo (*Dendrocalamus latiflorus*) *de novo* shoot organogenesis

**DOI:** 10.1093/hr/uhad223

**Published:** 2023-11-08

**Authors:** Nannan Wang, Wenjia Wang, Yang Cheng, Changyang Cai, Qiang Zhu

**Affiliations:** Basic Forestry and Proteomics Center (BFPC), College of Forestry, HaiXia Institute for Science and Technology, Fujian Agriculture and Forestry University, Fuzhou 350002, China; Basic Forestry and Proteomics Center (BFPC), College of Forestry, HaiXia Institute for Science and Technology, Fujian Agriculture and Forestry University, Fuzhou 350002, China; Basic Forestry and Proteomics Center (BFPC), College of Forestry, HaiXia Institute for Science and Technology, Fujian Agriculture and Forestry University, Fuzhou 350002, China; Basic Forestry and Proteomics Center (BFPC), College of Forestry, HaiXia Institute for Science and Technology, Fujian Agriculture and Forestry University, Fuzhou 350002, China; Basic Forestry and Proteomics Center (BFPC), College of Forestry, HaiXia Institute for Science and Technology, Fujian Agriculture and Forestry University, Fuzhou 350002, China

## Abstract

Bamboo is an important non-timber forest product and is well-known for its reluctance to regenerate. Recently we have established a *de novo* shoot organogenesis (DNSO) protocol in Ma bamboo (*Dendrocalamus latiflorus*) and revealed the transcriptomic dynamics during Ma bamboo regeneration, which suggested the potential roles of Ma bamboo microRNAs (DlamiRNAs) in this process. However, how DlamiRNAs regulate bamboo DNSO is poorly understood. Here we performed integrated analysis with sRNAome, degradome, and transcriptome sequencing by using samples covering the four stages of the bamboo DNSO process. A total of 727 DlamiRNAs showed differential expression during the bamboo DNSO process, and the core DlamiRNA–DlamRNA- mediated regulatory networks for bamboo DNSO were constructed. Based on the results, DlamiR156 was selected for further functional characterization of its potential roles in bamboo DNSO. Transgenic bamboos with increased DlamiR156 levels exhibited an enhancement in their regeneration efficiency. Conversely, when DlamiR156 levels were downregulated, the regeneration efficiencies of transgenic bamboos decreased. Our findings show that the DlamiRNA-mediated regulatory pathways are significant in the process of bamboo regeneration and will contribute to our understanding of the molecular mechanisms governing plant organogenesis in a more comprehensive manner.

## Introduction

Compared with animals, plants have remarkable regenerative abilities due to the property of developmental plasticity of their cells. Plant regeneration is usually achieved through somatic embryogenesis (SE) or *de novo* shoot organogenesis (DNSO). Compared with SE, the DNSO pathway is simpler and more robust, and therefore more widely applied in practical biotechnology breeding, such as plant genetic transformation, genome editing, and micropropagation. DNSO relies on the activation of regeneration-initial cells and the acquisition of competency, which are regulated by a number of external and internal factors that guide the formation and development of the new stem cell niche [1]. The DNSO technique has been applied in various plant species, and the procedure always requires customized development or fine tuning on a case-by-case basis, owing to the considerable variation in embryogenesis responsiveness among different species, and even between different genotypes within a particular species [1]. The development of predicted and routine DNSO remains a big challenge. The molecular mechanism controlling this process remains to be elucidated, and the important genetic regulators of DNSO need to be identified.

Although it has not been extensively investigated, emerging evidence supports the idea that microRNAs (miRNAs) are crucial genetic regulators in plant regeneration regulatory networks [1, 2]. miRNAs, which have 19–24 nucleotides, are non-coding small RNA regulatory molecules that act as master regulators of plant development [3]. With the development of miRNA detection techniques, such as high-throughput miRNA sequencing technology and bioinformatic analysis tools, thousands of miRNAs have been identified in different plant species, and a number of differentially expressed miRNAs (DEMs) involved in plant regeneration have been identified based on bioinformatic prediction or sequencing datasets in various plant species, such as rice [4], *Arabidopsis* [5], *Citrus* [6], *Larix leptolepis* [7], maize [8], cotton [9], and Ma bamboo (*Dendrocalamus latiflorus*) [10].

Plant miRNAs perform their repressive regulation in two major ways: transcript cleavage and translation repression. Most mRNA cleavage occurs when the target mRNA sequence is closely matched to the miRNA [2]. Although numerous miRNAs were identified through sequencing analysis, the role of specific miRNA in plant regeneration remains to be elucidated. The application of target mimicry technologies, such as target MIMICs (MIMs) and short tandem target MIMICs (STTMs) [11, 12], has presented effective means for studies on hindering the activity of endogenous mature miRNAs [13], and for facilitating functional studies of numerous miRNAs in plant regeneration. Specifically, it was reported that miR408 regulates *nudix hydrolase* 23 (*DlNUDT23*), which functions in mediating N^6^-methyladenosine (m^6^A) modification and influencing RNA homeostasis and cell cycle gene expression during early SE in longan [14]. In *Arabidopsis*, miR394 strongly enhances SE when expressed ectopically together with *WUSCHEL* (*WUS*) in the recalcitrant *Ler Arabidopsis* accessions [15], while miR393 inhibits shoot regeneration via repressing the auxin receptor *TIR1* [16]. Similarly, miR160 negatively controls *Arabidopsis* shoot regeneration capacity through its target gene *ARF10*, which mediates regulation of shoot meristem-specific genes *CLAVATA3*, *CUP-SHAPEDCOTYLEDON-1/2*, and *WUS* [5]. In addition, LamiR166a from *Larix leptolepis* [7], Zma-miR528 from maize, and miR167 from cotton [17] were also reported to be essential for plant somatic embryogenesis, and modulating these miRNA expression patterns substantially altered the plant’s regeneration capacity.

The miR156 family, which is one of the most conserved miRNA families in plants, targets various *SQUAMOSA PROMOTER BINDING PROTEIN-LIKE* (*SPL*) transcription factors and regulates multiple biological processes [18]. miR156 has been shown to act as an intrinsic regulator in plant regeneration [19]. In *Arabidopsis*, miR156–*SPL9/SPL15* modules repress the downstream B-type *Arabidopsis* response factors (*ARR*s) *ARR1*, *ARR2*, *ARR10*, and *ARR12*, which function in the cytokinin signaling pathway and reduce shoot regeneration efficiency [19]. In citrus, miR156 accumulated to abundant levels in the embryogenic callus [6] and the CsmiR156–*CsSPL3/CsSPL14* module regulated the somatic embryogenesis potential of citrus callus. The expression of CsmiR156 is regulated by its upstream activators *AGAMS-LIKE15* (*CsAGL15*) and *FUSCA3* (*CsFUS3*), while its *CsSPL* targets inhibit the expression of Csi-miR172d and promote the expression of the targets of *Egr1* (*CsTOE1.1* and *CsTOE1.2*), which inhibit the expression of starch synthesis and transport-related genes, and thus reduce amyloplast and starch accumulation in the callus cells that are required for SE [20]. There have been great achievements in our understanding of miRNA’s function during plant regeneration, but the temporal and spatial expression as well as the regulatory mechanisms of miRNAs in different species vary greatly, and the regulatory pathway of miRNA remains to be systematically studied, especially for some economically and ecologically important forestry species, such as bamboo.

Bamboo is well known for its fast growth and lignocellulose-abundant characters, and it has become one of the most important non-timber forest resources globally [21]. Due to its long and irregular flowering habit, it is quite difficult to generate new bamboo germplasm through classical breeding [22–24]. There have been several reports describing bamboo improvement through genetic engineering [25–27], but for most bamboo species regeneration is quite difficult, and shoot organogenesis efficiency remains to be improved. Recently, we have successfully established an efficient shoot organogenesis system in Ma bamboo (*D. latiflorus*) [26], and found that Ma bamboo was regenerated through indirect organogenesis instead of somatic embryogenesis [28]. Time-course transcriptome analyses revealed that many DlamiRNA-related transcripts substantially altered their expression patterns during the DNSO process, indicating that DlamiRNAs may act as important regulators in bamboo DNSO [28]. Nevertheless, the dynamics of DlamiRNA expression and the molecular networks mediated by DlamiRNAs during bamboo shoot organogenesis have yet to be elucidated. Additionally, there is currently a lack of functional characterization for any specific DlamiRNA involved in regulating bamboo regeneration.

In this study we present an integrated analysis utilizing sRNAome sequencing, degradome sequencing, and transcriptome sequencing to unravel the underlying mechanisms governing DNSO in Ma bamboo. We identified a total of 3716 DlamiRNAs during bamboo shoot organogenesis, comprising 15 known DlamiRNAs and 3701 novel DlamiRNAs. Notably, a comprehensive analysis revealed that 727 DlamiRNAs exhibited differential expression patterns throughout the process. Subsequently, we constructed a core regulatory ‘DlamiRNA–DlamRNA’ network for Ma bamboo regeneration. Moreover, we observed a significant over-representation of the DlamiR156-*DlaSPL* module, indicating its crucial role in this process. We created transgenic bamboo lines with a short tandem target mimic for DlamiR156 (STTM156) and overexpressed pre-DlamiR156 (miR156OE). Remarkably, we observed that transgenic bamboos with upregulated DlamiR156 levels exhibited enhanced regeneration efficiencies, while those with downregulated DlamiR156 levels displayed decreased regeneration efficiencies. These findings provide additional evidence supporting the hypothesis that DlamiR156 plays a crucial role not only in SE as reported previously, but also in the DNSO of bamboo. In summary, the integrated analysis in this study provides insights into the comprehensive DlamiRNA–DlamRNA based transcriptomic dynamics and regulatory network components associated with bamboo shoot organogenesis, and provides an extensive resource for functional genomics studies on DNSO in bamboo.

## Results

### DlamiRNA expression patterns during Ma bamboo shoot organogenesis

**Figure 1:**
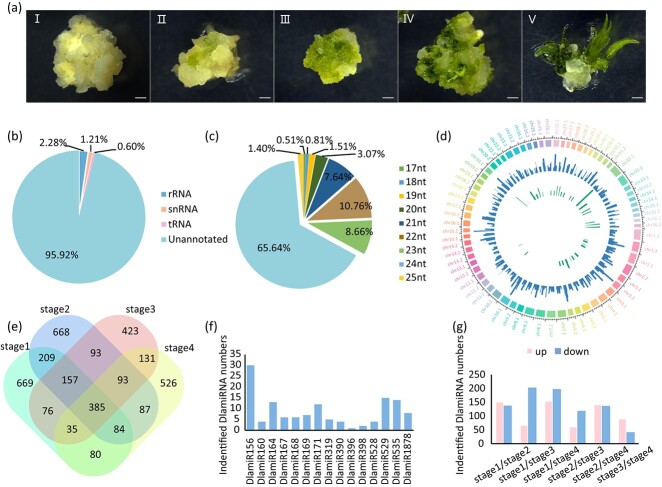
Analysis of the sRNAome in Ma bamboo. **a***De novo* shoot organogenesis process. Calluses were transferred from callus multiplication medium to shoot induction medium, and the developmental status of the samples was recorded. Scale bar = 1 cm. **b** Distribution of sequence types in sRNAome analysis. Proportions of annotated and unannotated sequences, further analyzed to identify DlamiRNAs, are shown. **c** Frequencies of DlamiRNAs with different lengths. **d** Mapping of identified DlamiRNAs to the Ma bamboo genome. The outer circular representation corresponds to chromosomes, and the height of the bars represents the read frequency of the mapped DlamiRNAs. Middle circular indicate novel DlamiRNAs, while inner circular represent known DlamiRNAs. **e** Venn diagram showing the overlap and unique sets of mature DlamiRNAs identified at different stages of shoot organogenesis. **f** Conserved DlamiRNA families, i.e. DlamiRNA families that are found across different species and are evolutionarily conserved. **g** Differential expression of DlamiRNAs (DEMs) during shoot organogenesis. Upregulated or downregulated DlamiRNAs at different stages of shoot organogenesis are presented.

Previously, the bamboo DNSO process was divided into five stages based on in-depth analysis at cellular levels, and the newly developing bamboo bud that originated from the callus was observed at stage 4 [28] (Fig. 1a). Therefore, the samples from the first four stages were harvested separately and used for small RNA library construction and sequencing, to capture the DlamiRNA expression dynamics during Ma bamboo DNSO and to identify the key DlamiRNAs involved in this process. In general, the small RNA data showed high reproducibility among different biological replicates and can reflect the continuity of the bamboo shoot organogenesis process (Supplementary Data Fig. S1a). In total, 233.42 million reads were generated from the 12 small RNA libraries, and 59.92 million clean reads were obtained after filtering and trimming, with >3.55 million per sample (Supplementary Data Table S1). The rRNAs, snRNAs, and tRNAs were removed from clean reads, and 95.92% of the unannotated sequences were used for DlamiRNA identification (Fig. 1b). In combination, 3716 DlamiRNAs (consisting of 15 known and 3701 novel DlamiRNAs) were detected. The identified DlamiRNAs exhibited a range of lengths, spanning from 17 to 25 nt. Among these, the most abundant DlamiRNA length was 24 nt, accounting for 65.54% of the total, followed by 22 nt, which accounted for 10.76% (Fig. 1c). The distribution profile of known and novel DlamiRNAs along each bamboo chromosome is illustrated in Fig. 1d.

To investigate the dynamics of DlamiRNA expression during Ma bamboo DNSO, the expression patterns of DlamiRNAs at individual stages were studied. A total of 1695, 1776, 1393, and 1421 mature DlamiRNAs were identified during stages 1–4, respectively (Fig. 1e, Supplementary Data Fig. S1b). Out of the identified DlamiRNAs, 385 mature DlamiRNAs were present throughout all stages of bamboo DNSO, while 669, 668, 423, and 526 mature DlamiRNAs were specifically expressed in stage 1 to stage 4, respectively (Fig. 1e). Among the identified DlamiRNAs, most were novel DlamiRNAs, while the known DlamiRNAs belonged to 15 miRNA families, including the miR156 family, which had 30 precursors (Fig. 1f). To identify DlamiRNAs potentially involved in bamboo DNSO, DEMs were further analyzed across the four stages, and a total of 727 DEMs were identified using a cutoff of *P*-value <.05 and |log_2_(FC)| > 1 (Fig. 1g, Supplementary Data Table S2). These DEMs exhibited upregulated or downregulated expression patterns across the different stages (Fig. 1g). To validate the expression patterns of DlamiRNAs obtained from small RNA sequencing, we conducted stem–loop RT–qPCR analysis, the results of which confirmed the reliability of the high-throughput sequencing (Supplementary Data Fig. S2). In summary, our time-course analysis effectively captures the distinct patterns of DlamiRNA expression throughout the stages of bamboo DNSO.

### Identification of DlamiRNA targets by degradome sequencing analysis

To identify the Ma bamboo DNSO DlamiRNA targets, we generated degradome libraries from the same stage of samples used for miRNA and transcriptome profiling. A total of 250.44 million reads were obtained after consecutive steps of filtering, and at least 43.16 million reads per sample were derived (Supplementary Data Table S3). By analyzing the degradome data, a total of 374 targets were identified for 203 DlamiRNAs, and 614 DlamiRNA–DlamRNA pairs were confirmed (Supplementary Data Table S4). We noticed that the maximum targets were obtained for members of the DlamiR156 family (59) (most of them belonging to the *SPL* family), followed by DlamiR164 (33), DlamiR319 (17), and DlamiR398 (10) (Supplementary Data Table S4).

**Figure 2:**
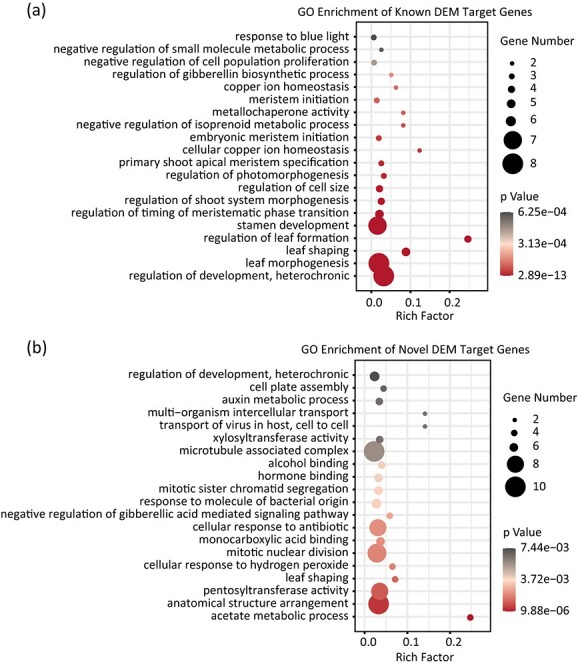
GO enrichment analysis of DEM target genes. **a** GO enrichment analysis of novel DEM target genes. **b** GO enrichment analysis of known DEM target genes.

To reveal the functions of the target genes, Gene Ontology (GO) enrichment analysis was performed. A total of 80 plant-known DlamiRNA targets were identified, and they were involved in multiple biological process related to plant regeneration, including regulation of cell size (GO:0008361) and embryonic meristem initiation (GO:0090421). In these GO terms, many previously reported regeneration-related genes were identified, such as *SPL14* and *SPL17*, which are involved in cell wall regeneration [29], and *NAC021* and *NAC098*, which function in shoot meristem formation [30] (Fig. 2a, Supplementary Data Table S5). A total of 1034 novel DEM target genes were also identified, and significantly enriched GO items were anatomical structure arrangement (GO:0048532), mitotic nuclear division (GO:0140014), and auxin metabolic process (GO:0009850) such as the auxin signaling pathway gene *ARF3* and the auxin biosynthesis gene *YUC3* (Fig. 2b, Supplementary Data Table S5), indicating that these biological processes are required for bamboo DNSO. Moreover, the results revealed that the common biological processes that were substantially enriched in both known and novel DEM target genes were regulation of development, heterochronic (GO:0040034) and leaf shaping (GO:0010358), such as *PCF6*, *SPL3*, and *SPL12*. These results suggested that DlamiRNAs may have a large impact on bamboo DNSO through regulating the abundance of its target genes.

### DlamiRNA–DlamRNA-downstream gene network during Ma bamboo shoot organogenesis

**Figure 3:**
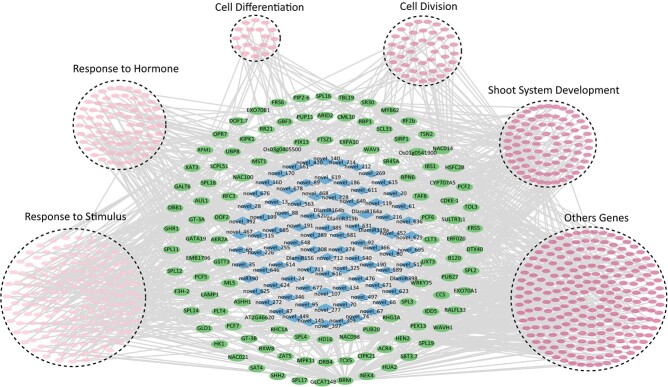
DlamiRNA–DlamRNA downstream gene network during Ma bamboo shoot organogenesis. Diamonds represents DEMs during shoot organogenesis. Circles represents target genes of DEMs, while circle with dotted lines represents downstream genes.

Alternation splicing of miRNAs results in large-scale changes in transcription. To investigate how DlamiRNAs regulate the downstream transcripts via its targets, we carried out correlation analysis with the datasets from sRNAome, degradome, and transcriptome analyses. Through this analysis, the interactions between DlamiRNAs and their target mRNAs were identified, and these interactions were further connected to downstream genes involved in Ma bamboo DNSO (Fig. 3, Supplementary Data Table S6). Based on the prediction, DEGs that interact with the DEM target genes during the shoot organogenesis process were divided into six groups: response to stimulus; response to hormone; cell differentiation; cell division; shoot system regulation; and others (Fig. 3, Supplementary Data Table S6). Notably, these results are consistent with the molecular events outlined in our prior report [28].

The results showed that multiple known DlamiRNAs, including DlamiR156 and DlamiR319, as well as novel DlamiRNAs such as novel_66, novel_70, novel_255, novel_346, novel_410, novel_468, and novel_636 target genes, participated in shoot organogenesis by interacting with multiple downstream genes that function in this process (Supplementary Data Fig. S3). Of these, the target genes of DlamiR156 were mainly *DlaSPL* transcription factors, and they may participate in shoot organogenesis in Ma bamboo by regulating the expression of *RCN1*, *BRI1*, *GI*, and *PID* (Supplementary Data Fig. S3). Besides, *RR21*, as the target gene of DlamiR156, plays a role in the cytokinin signaling pathway (Supplementary Data Fig. S3). The target genes of DlamiR319 were for TCP family proteins (*PCF6*, *PCF7*, *PCF8*, *GLO1*). In addition to *GAM1* and *SL1*, which were involved in regulating shoot system development, other genes downstream of *TCP* mainly play roles by affecting stress-related genes. *BRM* was the target gene of novel_410, and most of its downstream genes were related to shoot system development (Supplementary Data Fig. S3).

The network reveals the complex regulatory relationships between DlamiRNAs, mRNAs, and downstream genes, shedding light on the coordinated molecular processes driving shoot organogenesis in Ma bamboo.

### A DlamiRNA–DlamRNA atlas during Ma bamboo shoot organogenesis

**Figure 4:**
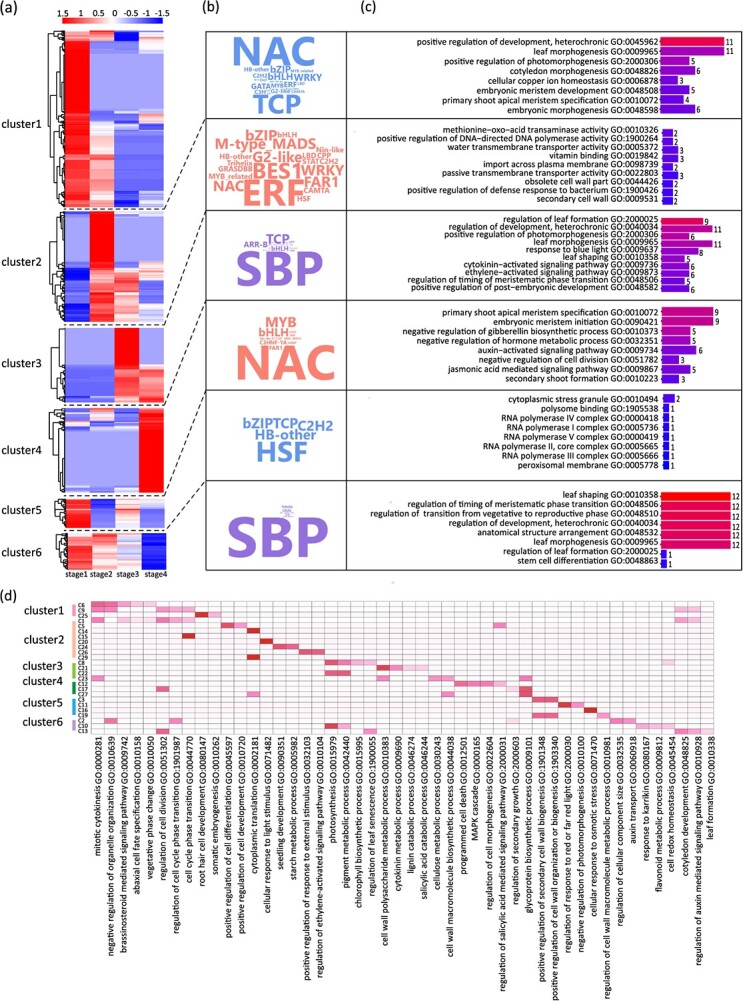
Correlation analysis of sRNAome, degradome, and transcriptome. **a***K*-means clustering analysis of 727 DEMs. **b** Enrichment analysis of target gene transcription factor families for each DEM cluster. Font size represents the degree of enrichment. **c** GO enrichment analysis of target genes for each DEM cluster. **d** Integration of transcriptome data with sRNAome and degradome analyses. Transcriptome clusters that exhibit either similar or opposite expression patterns compared with the DlamiRNA target genes were selected.

To further understand the expression dynamics of DlamiRNA and DlamRNA and to reveal their expression correlations, we performed integrated analysis of the sRNAome, degradome, and transcriptome data reflecting the dynamics during bamboo DNSO. The expressed DlamiRNAs and DlamRNAs were clustered based on the stage-specific changes in their expression during the bamboo DNSO process. Results from *K*-means clustering analysis showed that six clusters (cluster 1 to cluster 6) were generated from the 727 DEMs during shoot organogenesis, and for each cluster, the enrichment of transcription factors that putatively act as the targets of DlamiRNAs, as well as gene GO enrichment, are presented (Fig. 4a, Supplementary Data Tables S7 andS8). Moreover, the transcriptome data were divided into 30 clusters (C1–30) according to their different expression patterns (Supplementary Data Fig. S4). Clusters that were the same or opposite to the DlamiRNA target gene expression pattern were selected for further analysis (Fig. 3d, Supplementary Data Table S9). We integrated published transcriptome data with sRNAome and degradome analysis to explore downstream genes affected by DlamiRNA–DlamRNA.

In Cluster 1, miRNAs have the highest expression levels at stage 1 (Fig. 4a), when the callus exhibits an undifferentiated cell mass status [28]. The known DlamiRNA families, such as DlamiR164b, DlamiR319b, and DlamiR398, as well as 67 novel DlamiRNAs, were overrepresented in this cluster (Fig. 4a, Supplementary Data Table S7), which indicates that these DlamiRNAs may mainly contribute to keeping the dedifferentiation of callus. The enriched transcription factors mainly belonged to the NAC and TCP families, which are potentially involved in the regulation of embryonic meristem development (GO:0048508), leaf morphogenesis (GO:0009965), and cellular copper ion homeostasis (GO:0006878) based on GO enrichment analysis (Fig. 4a, Supplementary Data Table S8). Their downstream genes related to mitotic cytokinesis, abaxial cell fate specification, vegetative phase change, and cell cycle phase transition are enriched, such as the previously reported *KIN4* [31], *CDC7* [32], *ARF3*, and *IAA4* [33] (Fig. 4b), suggesting that these DlamiRNAs may function in cell proliferation and that cell fate may be determined at this stage. In Cluster 2, the novel DlamiRNAs are substantially enriched at stage 2 (Fig. 4a, Supplementary Data Table S7), when the callus was transferred to the shoot induction medium and adventitious shoot primordia emerge [28]. The downstream putative targets include transcription factors belonging to ERF, BES1, WRKY, bZIP, and so on (Fig. 4a, Supplementary Data Table S8), which were involved in transporter activity as well as cell wall-related regulation (Fig. 4a, Supplementary Data Table S8). At this stage, their downstream genes related to cell differentiation and cell development, somatic embryogenesis, light stimulus, and seedling development, such as *SCC2*, *LAF1*, and *PYL2*, were substantially enriched (Fig. 4b), suggesting that these genes are potentially important during the transition from cell dedifferentiation to differentiation. In Cluster 3, known DlamiRNAs such as DlamiR156 and DlamiR319a, as well as 29 novel DlamiRNAs, were highly expressed at stage 3 (Fig. 4a, Supplementary Data Table S7), when the shoot primordia initiated their formation [28]. The putative downstream targets of these DlamiRNAs were transcription factors belonging to the SBP, TCP, and ARR-B families, such as FTSH1, LHBC, NAC030, and NAC007, which function in the regulation of leaf formation (GO:2000025), cytokinin-activated signaling pathway (GO:0009736), and regulation of development, heterochronic (GO:0040034) (Fig. 4a, Supplementary Data Table S7), and they may regulate the expression of downstream genes related to photosynthesis and cell wall-related metabolic process (Fig. 4b). In Cluster 4, DlamiR164a and 38 novel DlamiRNAs were overrepresented at stage 4, when the newly developing buds appear (Fig. 4a, Supplementary Data Table S7). The putative downstream DlamiRNA targets are transcription factors belonging to the *NAC*, *MYB*, and *bHLH* gene families, which function in regulation of primary shoot apical meristem specification (GO:0010072) and negative regulation of gibberellin biosynthetic process (GO:0010373) (Fig. 4a, Supplementary Data Table S8). In Cluster 5 and Cluster 6, DlamiRNAs may function in several developmental stages, indicating that they are constantly active during the regeneration process. Transcription factors belonging to the SBP, HSF, MYB, NAC, and TCP families, which function in RNA transcription and cell division and differentiation, were substantially enriched (Fig. 4a, Supplementary Data Table S7). Correspondingly, their downstream genes related to development, phytohormone response, metabolic process, and stress response were overrepresented (Fig. 4b).

In summary, the DlamiRNA–DlamRNA-downstream gene modules that participated in bamboo DNSO were constructed, and the data presented here also suggested the DlamiRNA-mediated dynamic and sequential transcription regulation that controls cell fate transition during this process.

### Expression patterns of DlamiR156 and prediction of its putative targets during bamboo shoot organogenesis

Of the known DlamiRNAs that were identified as putative regulators for bamboo DNSO, the DlamiR156 family was the most prominent, and a total of 30 putative DlamiR156 precursors were identified (Supplementary Data Table S10). Furthermore, by exploring the degradome data, we found that DlamiR156 exhibited the highest number of targeted genes, encompassing a total of 59, out of which 50 genes were classified as *DlaSPL* transcription factors (Supplementary Data Table S4). Due to the inhibitory effect of DlamiRNA on downstream target genes, we selected DlamiR156 and its target genes, which exhibited contrasting expression patterns, for additional verification. Through qPCR analysis, we demonstrated that DlamiR156 exhibits a differentially expressed pattern during bamboo DNSO, and that there is a significant increase in the expression level of DlamiR156 during the third stage of shoot organogenesis compared with the initial stage (Supplementary Data Fig. S2).

**Figure 5:**
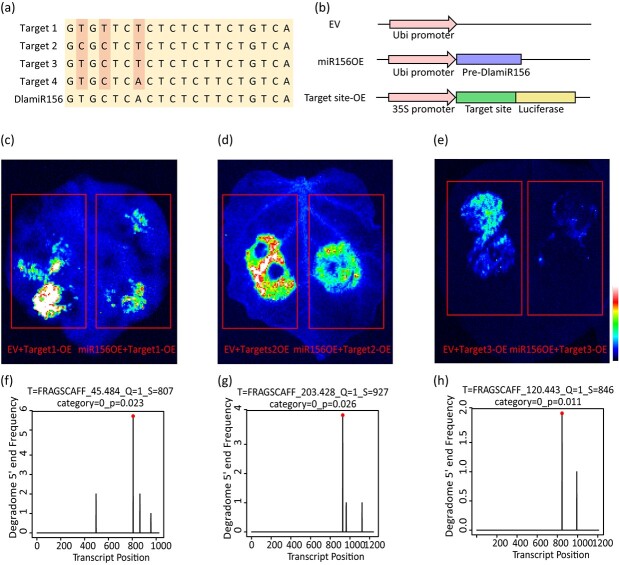
Verification of DlamiR156 and its target gene sites. **a** Sequences of DlamiR156 and its target gene sites. **b** Information on the empty vector (EV), miR156OE, and target site overexpression vectors used for transient expression in tobacco. **c**–**e** Co-infiltrated leaf after infiltration observed using a Tanon 5200 multi-imaging system. **f**–**h** Cleavage positions predicted from degradome data. Dots indicate the strongest signal.

The 50 *DlaSPL* genes that were identified from degradome sequencing showed four different types of DlamiR156 targeting sequences (Fig. 5a). Subsequently, vectors expressing DlamiR156 and putative target sequences were constructed to validate the interaction between DlamiRNA and target genes (Fig. 5b). After expression of these vectors in tobacco leaves, the expression of DlamiR156 substantially attenuated the luciferase activity at putative target sites (Fig. 5c–e, Supplementary Data Fig. S5), which is consistent with the results from the T-plot of the degradome (Fig. 5f–h). The results above indicated that DlamiR156 is potentially involved in bamboo DNSO by cleaving the identified *DlaSPL*-targeted genes.

### DlamiR156 positively participates in the shoot organogenesis process

**Figure 6:**
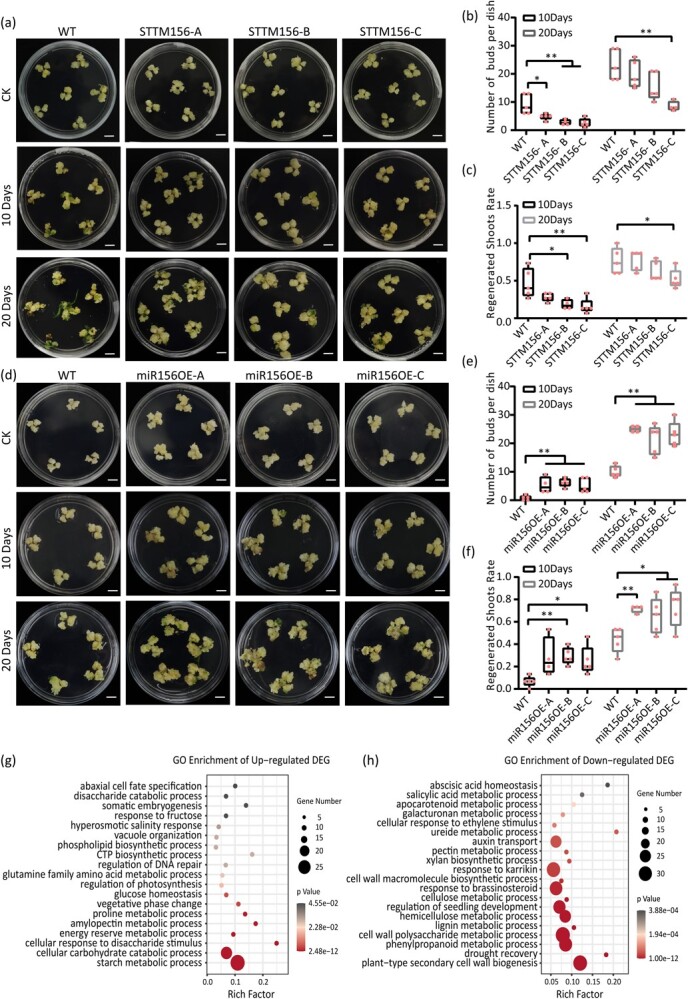
Function characterization of DlamiR156 during Ma bamboo shoot organogenesis. **a** Phenotypes of WT and STTM156 transgenic line calluses after shoot induction for 0, 10, and 20 days. Scale bar = 1 cm. **b** Statistical analysis of newly generated shoots per dish in WT and STTM156 transgenic lines after shoot induction for 0, 10, and 20 days. Error bars represent standard errors from at least three replicates (*P < .05, **P < .01). **c** Regeneration efficiencies of WT and STTM156 transgenic lines after shoot induction for 0, 10, and 20 days. Error bars represent standard errors from at least three replicates (*P < .05, **P < .01). **d** Phenotypes of WT and miR156OE transgenic line calluses after transfer to shoot induction medium for 0, 10, and 20 days. Scale bar = 1 cm. **e** Statistical analysis of newly generated shoots per dish in WT and miR156OE transgenic lines after shoot induction for 0, 10, and 20 days. Error bars represent standard errors from at least three replicates (**P < .01). **f** Regeneration efficiencies of WT and miR156OE transgenic lines after shoot induction for 0, 10, and 20 days. Error bars represent standard errors from at least three replicates (*P < .05, **P < .01). **g** GO enrichment analysis of upregulated DEGs between WT and STTM156 transgenic lines. **h** GO enrichment analysis of downregulated DEGs between WT and STTM156 transgenic lines.

The mature DlamiR156 sequence from Ma bamboo is conserved with that in *Arabidopsis*, rice and wheat (Supplementary Data Fig. S6a). A total of 30 DlamiR156 precursor loci were mapped to chromosomes based on sRNAome sequencing, and their secondary structures are characterized by a classic stem-loop configuration (Supplementary Data Fig. S6b and c). To investigate the biological function of DlamiR156 during shoot organogenesis in Ma bamboo, we applied the STTM-mediated silencing technology to repress DlamiR156 function in bamboo. A vector containing two imperfect DlamiR156 binding sites separated by an 88-bp spacer was constructed (Supplementary Data Fig. S6d), and introduced into Ma bamboo via the established bamboo transformation protocol [26]. STTM156 transgenic lines were successfully generated. Further molecular verification by stem–loop RT–qPCR confirmed the downregulation of DlamiR156 in STTM156 lines (Supplementary Data Fig. S6e). Moreover, miR156OE bamboos were also generated (Supplementary Data Fig. S6f). In order to assess the influence of miR156 on bamboo shoot organogenesis, initial wild-type (WT) callus samples, matching the status of their corresponding STTM lines or miR56OE lines, were carefully chosen for further shoot induction experiments. Following 10 days of shoot induction, WT callus displayed distinct green spots, whereas the STTM156 transgenic lines showed significantly fewer spots compared with WT (Fig. 6a). By the 20th day, WT callus had already produced leaves, whereas STTM156 lines were still at the early stage of shoot emergence. The occurrence of green spots and the rate of shoot regeneration were monitored and subjected to statistical analysis (Fig. 6b and c). Consistently, the miR156OE transgenic lines exhibited a contrasting phenotype. Compared with the WT, the miR156OE transgenic lines displayed an increased number of green spots and a higher shoot regeneration rate after 10 and 20 days of culture (Fig. 6d–f).

In order to investigate the mechanism by which DlamiR156 regulates shoot organogenesis, we conducted transcription analysis (RNA-seq) on both WT and STTM156 transgenic lines. A total of 4422 differentially expressed genes (DEGs) were identified using a cutoff of *P*-value <.05 and |log_2_(FC)| > 0.5 between the WT and STTM156 transgenic lines. Among these DEGs, 1602 were upregulated and 2820 were downregulated in the STTM156 transgenic lines (Supplementary Data Table S11, Supplementary Data Fig. S7a). Results from GO enrichment analysis showed that upregulated genes were mainly involved in somatic embryogenesis (GO:0010262), vegetative phase change (GO:0010050), and proline metabolic process (GO:0006560) (Fig. 6g, Supplementary Data Table S12), while the upregulated genes were involved in plant-type cell wall organization (GO:0009664) and regulation of seedling development (GO:1900140). They also participated in phytohormone pathways, such as cellular response to ethylene stimulus (GO:0071369), salicylic acid metabolic process (GO:0009696), auxin transport (GO:0060918), response to brassinosteroid (GO:0009741), and abscisic acid homeostasis (GO:1902265) (Fig. 6h, Supplementary Data Table S12). These results showed that DlamiR156 might trigger multiple biological processes that contribute to bamboo regeneration.

Many reports showed that DlamiR156 exerts its functions by repressing the expressions of its downstream *SPL* transcription factors. To identify the DlamiR156–*DlaSPL* module that potentially functions in bamboo shoot organogenesis, firstly genome-wide analysis of the *DlaSPL* genes in Ma bamboo was performed, and 102 *DlaSPL* genes were identified (Supplementary Data Table S13). By integrating analysis of the degradome sequencing data, RNA-seq data during shoot organogenesis process, and RNA-seq data between WT and STTM156 transgenic lines, six *DlaSPL* genes were not only upregulated in STTM156 transgenic lines but also showed differential expression during shoot organogenesis (Supplementary Data Fig. S7b and c, Supplementary Data Table S13). Notably, of the six identified *DlaSPL*s, *DlaSPL42* and *DlaSPL96* not only showed opposite expression patterns compared with the expression of DlamiR156 during shoot organogenesis, but were also downregulated in the miR156OE transgenic lines and upregulated in the STTM156 transgenic lines, respectively, indicating that these two *DlaSPL*s likely have significant roles in the process of bamboo shoot organogenesis (Supplementary Data Fig. S8).

In summary, our results indicate that DlamiR156 may play a positive regulatory role in Ma bamboo DNSO.

## Discussion

DNSO is a well-orchestrated process that is primarily controlled by transcription and post-transcriptional regulators. Hierarchical transcriptional regulation, influenced by diverse regulators including those involved in epigenetics, such as miRNA, has been extensively studied in the model plant *Arabidopsis*, and numerous regulators have been identified and utilized in enhancing regeneration efficiency in plants [1]. However, understanding of the regeneration process in non-timber plants, such as bamboo, remains limited. Recently, we conducted a pioneering study that captured the transcriptome dynamics during bamboo shoot regeneration, which shed light on the potential roles of DlamiRNAs in bamboo shoot organogenesis [28]. Nonetheless, a comprehensive understanding of DlamiRNA populations and their regulatory networks controlling bamboo shoot organogenesis remains to be achieved. The release of genomic information [34] and the establishment of several genetic manipulation tools in Ma bamboo [26, 27] facilitate its bioinformatic analysis and the functional characterization of miRNAs. Here we generated time-course profiles of DlamiRNAs, and integrated them with degradome and transcriptome data to identify the corresponding target transcripts during bamboo shoot regeneration. Our findings, particularly those relating to the functional significance of DlamiR156, offer valuable resources for identifying regeneration efficiency regulators and for comprehensive investigations into the molecular mechanisms underlying bamboo regeneration.

### miRNAs are important regulators of the bamboo shoot organogenesis process

MiRNAs play critical roles as post-transcriptional regulators of gene expression, and they are increasingly recognized as essential regulators of plant regeneration processes. In bamboo, although a few miRNA resources have been released [35–38], no research has been conducted on the regeneration process of bamboo, and up to now no miRNA was functionally characterized in bamboo. In this study, 3717 DlamiRNAs were identified during bamboo shoot organogenesis. Among them, 15 DlamiRNAs were known and showed homology to miRNAs found in other species, and the remaining 3701 DlamiRNAs were novel and likely to be specifically expressed in Ma bamboo. Diverse levels of miRNAs may exist across various tissues or stages of development. The number of miRNAs identified in this study exceeded the numbers found in other studies. The large number of DlamiRNAs identified here suggested the complexity of the regulatory functions of DlamiRNAs in the control of bamboo DNSO. Moreover, we noticed that only a small proportion of the annotated DlamiRNAs in the plant were expressed during organogenesis (Fig. 1e). This finding indicated that only a specific subset of DlamiRNAs potentially play a role in regulating gene expression during bamboo DNSO, while the remaining DlamiRNAs might be involved in different tissues or other biological process. To screen DlamiRNAs that may play crucial roles in different DNSO stages, we analyzed DlamiRNA expression dynamics during the whole process, and identified the differentially expressed DlamiRNAs (Fig. 3). At present, several known DlamiRNAs, including DlamiR156, DlamiR171, DlamiR167, DlamiR408, and DlamiR394, have been found to be involved in plant regeneration via SE in most cases [14, 15, 17, 39]. Consistent with these findings, several conserved DlamiRNAs, including DlamiR156, were also identified as potential regulators in bamboo DNSO (Supplementary Data Fig. S3), suggesting that these DlamiRNAs may have a conserved and universal function in controlling plant regeneration. Additionally, our study also revealed that certain known DlamiRNAs, such as DlamiR164, may also be potentially involved in plant DNSO (Supplementary Data Fig. S3). Therefore, the dataset generated here can serve as a valuable resource for exploring unidentified regulators involved in plant regeneration.

### DlamiRNA–DlamRNA regulatory networks act as a key player during the shoot organogenesis process

One main goal for this study was to investigate the regulatory roles of DlamiRNAs and construct a DlamiRNA–DlamRNA gene network that regulates the bamboo DNSO process by coordinated analysis of sRNAome, degradome, and transcriptome data. A total of 727 DlamiRNAs showed differential expression during the process of bamboo DNSO, involving 203 DlamiRNAs and 374 target genes (Fig. 3, Supplementary Data Table S4). Moreover, by analyzing the time-course DlamiRNA and DlamRNA expression dynamics, we generated a hierarchical transcriptional regulatory network that drives bamboo shoot organogenesis (Fig. 3). An analysis of mRNA expression clusters compared with the DlamiRNA expression clusters identified a potential role of specific DlamiRNA expression clusters in regulating the expression of DlamRNAs specific to a certain stage during bamboo shoot organogenesis (Fig. 4). These findings imply that DlamiRNAs likely possess distinct roles throughout different stages of Ma bamboo organogenesis. Similar observations have been reported in the context of embryogenesis processes [40]. Gene expression during bamboo shoot organogenesis is sequential, reflected by the expression patterns of DEMs at different stages (Fig. 3). Certain DlamiRNAs and their corresponding targets are expressed at different stages, indicating their potential stage-specific roles. By clustering the DlamiRNA–mRNA modules based on their expression dynamics at various stages, we gained a valuable opportunity to investigate the functionality and cooperative roles of these modules in a unique manner. Therefore, it is possible that the incorporation of multiple stage-specific DlamiRNAs may further enhance bamboo regeneration efficiency compared with using a single DlamiRNA as a booster. In addition, the hierarchical DlamiRNA expression expands our comprehension of the molecular regulatory mechanism underlying the process of bamboo shoot organogenesis. It also facilitates the study of gene function for each individual factor.

### A conserved role for the DlamiR156–*DlaSPL* pathway during bamboo shoot organogenesis

DlamiR156 is one of the most conserved miRNAs in plants, and its expression gradually decreases with the age of the plant [41]. Recent studies have also identified miR156 as an important factor in regulating plant somatic embryogenesis in citrus [20] and shoot regenerative capacity in *Arabidopsis* [19]. miR156 interacts with different *SPL* targets to function in different plant biological processes [42]. In *Arabidopsis*, miR156 regulates its downstream *SPL9/SPL15* transcription factors, which control the B-type ARRs (arabidopsis response regulators) in the cytokinin signaling pathway, thereby affecting adventitious shoot formation [19]. In this study, we confirmed the function of DlamiR156 in Ma bamboo shoot regeneration through overexpression and suppression of DlamiR156 (Fig. 6). Notably, Ma bamboo shoot regeneration occurs via the DNSO pathway, instead of SE [28]. The strong phenotypes due to altered DlamiR156 expression further confirm that DlamiR156 is required for proper developmental timing of gene expression programs during bamboo DNSO. As reported in other studies, *SPL* factors may also act as targets of DlamiR156 during Ma bamboo shoot organogenesis. To identify the *DlaSPL* genes that are potentially involved in this process, we performed integrated analysis of the DEGs between WT and STTM156 lines, DEGs from shoot organogenesis, and degradome data, and found that *DlaSPL42* and *DlaSPL96* genes were differentially expressed during bamboo shoot organogenesis (Supplementary Data Fig. S8) and upregulated in STTM156 transgenic lines, but downregulated in miR156OE transgenic lines (Supplementary Data Fig. S8). Moreover, they can be cleaved by DlamiR156 (Fig. 5). These results strongly suggested that *DlaSPL42* and *DlaSPL96* may act as the downstream targets of DlamiR156, and control bamboo shoot organogenesis. Future studies will address the regulatory mechanisms of DlamiR156 in controlling Ma bamboo shoot organogenesis, and whether controlled regulation of the DlamiR156–*DlaSPL42/96* module might be used for improving the regeneration abilities of recalcitrant plants. Furthermore, it is important to note that the regulation of bamboo DNSO by miR156 is intricate, and we cannot rule out the possibility that miR156 may interact with other candidate targets, such as *NAC021* and *NAC098* identified in this study, to influence the process. To address this important question, we plan to conduct separate, dedicated studies in the future.

## Conclusion

In conclusion, we have provided a comprehensive dataset revealing the time-course of DlamiRNA–DlamRNA expression patterns and generated the transcriptional regulatory network during the process of bamboo DNSO, and for the first time we have shown the function of DlamiRNA in bamboo regeneration. The described resources and phenotypes offer multiple avenues to delve deeper into the characterization of how DlamiRNA-mediated repression of transcripts, including those encoding transcription factors, contributes to the process of bamboo DNSO.

## Materials and methods

### Plant materials and growth conditions

Ma bamboo was regenerated based on our previously established protocol [26]. Briefly, Ma bamboo calluses were induced from stems on callus induction medium containing 30 g/l sucrose, 8 mg/l 2,4-dichlorophenoxyacetic acid (2,4-D), 0.5 mg/l indole butyric acid, and 4.2 g/l Phytagel. The collected callus was amplified on callus multiplication medium containing 3/4 MS, 30 g/l sucrose, 3 g/l sorbitol, 250 mg/l polyvinylpyrrolidone, 2 mg/l 2,4-D, and 4.2 g/l Phytagel. Then, the healthy callus was transferred to shoot induction medium containing MS medium, 30 g/l sucrose, 2 mg/l 6-benzylamino purine, 0.5 mg/l NAA, and 4.2 g/l Phytagel. The calluses were transferred onto the same fresh medium for subculture every month, until transgenic plants were obtained. All samples were grown on shoot induction medium for 0, 15, 21, 30, and 45 days and were collected as previously reported [28]. Callus induction and amplification were performed in dark conditions, while shoot induction was performed in a light environment with light intensity of 60–70 μmol/m^2^/s and photoperiod of 16 h light/8 h dark. The entire callus subculture process was performed at a temperature of 26 ± 2°C.

### Small RNA library construction and sequencing

Total RNA was extracted from calluses at the first four stages of the shoot organogenesis process. TRIzol™ reagent (Thermo, 15 596 026) was used for extraction, followed by purification. Two micrograms of RNA of per sample, consisting of three biological replicates, were utilized to construct small RNA libraries. Initially, adapters were added to the 3′ and 5′ ends of the sequences, and reverse transcription was employed to synthesize the first chain. Subsequently, PCR amplification was performed, and the resulting PCR products were purified. The small RNA library was constructed using the NextSeq CN500 sequencing platform. Raw sequences underwent quality control steps (qualified_quality_phred 30, unqualified_percent_limit 50 and N_base_limit 10), we used Trimmomatic software to remove adapters, and clean reads were obtained after filtering. Annotated small RNAs were removed, and Bowtie software was utilized to filter out potential mRNA fragments [43]. miRDeep2 software was employed to identify discovered small RNAs and detect novel small RNAs [44]. The chromosome localization circle of DlamiRNA precursors was visualized using TBtools [45]. DESeq2 software was used to identify differentially expressed DlamiRNAs, with a *P*-value cutoff of <.05 [46].

### Degradome library construction and analysis

Samples from the first four stages of shoot organogenesis were harvested and used for RNA extraction as previously reported [28]. Twenty micrograms of RNA per sample was used for degradome library construction. mRNA passing the quality assessment (qualified_quality_phred 30, unqualified_percent_limit 50 and N_base_limit 10) was captured using magnetic beads. The captured mRNA was mixed with 3′ and 5′ adapters and biotinylated random primers, and then subjected to reverse transcription followed by PCR amplification. The degradome libraries were constructed using the Illumina HiSeq 2500 sequencing platform. ACGT101-DEG software was employed for miRNA data analysis (LC Sciences, Houston, TX, USA). Typically, miRNA cleavage sites are found in the 10th–11th nucleotide region. The software locates the cleavage site in the mRNA and extracts potential complementary regions upstream of the 13-nucleotide site. This sequence is then input into a small RNA cleavage target-finding program to enhance the accuracy of target identification. Target gene prediction was performed using the CleaveLand program. After filtering the raw data, the alignable sequences were compared with the cDNA database sequence of the sequenced species to generate a degradome density file. GSTAr software was utilized to predict the mRNA sequences of target genes that paired with the small RNA sequences from Ma bamboo [47]. The predicted DlamiRNA target genes were combined with the mRNA in the degradome density file to identify common mRNAs as miRNA target genes. The degradation peak classification and scores were recorded, and t-plots were utilized to generate predictive results. To visualize the DlamiRNA target gene network, we used the STRING database (https://string-db.org/) with a minimum required interaction score of 0.4 to predict the downstream genes of miRNA target genes, and then Cytoscape software was employed [48].

### qPCR analysis and gene verification

To validate the small RNA sequencing, DlamiR156, DlamiR319b, and DlamiR398 were selected for stem–loop RT–qPCR [49]. miRNA was reverse-transcribed using SuperScript™ III Reverse Transcriptase (Thermo Fisher, 18-080-044) with U6snRNA as the internal reference gene, and fluorescent quantitative analysis was performed using SYBR Premix Ex Taq (Takara, RR390Q). To verify the results of transcriptome sequencing, DlamiR156 and its target genes, including *DlaSPL42* and *DlaSPL96*, were selected using the quantitative real-time polymerase chain reaction [qPCR, using NovoScript Plus All-in-one 1st Strand cDNA Synthesis SuperMix (gDNA Purge)] (Novoprotein, 05227808) for cDNA synthesis with bamboo *actin* as the internal reference gene and SYBR Premix Ex Taq (Takara, RR390Q) for fluorescent quantitative analysis. The specific primers of DlamiRNA and chosen genes are listed in Supplementary Data Table S14. The relative abundance of each gene was calculated using the 2^−∆∆Ct^ method, with three replicates for each reaction. The luciferase reporting system was used to verify the interaction between DlamiRNA and downstream genes as previously reported [36]. The target gene sequence was ~200 bp in length, including the DlamiRNA targeting cleavage site, and was inserted into a vector containing luciferase. The vector with both DlamiRNA and the target gene was then transferred to *Agrobacterium*, and injected into tobacco leaves for observation using the Tanon 5200 multi-imaging system.

### Vector construction, plant transformation, and phenotype observation

The STTM156 vector consists of a duplicated 35S promoter and two synthetic DlamiR156 short sequences joined together by 88 nucleotides as reported previously [13]. On the other hand, the miR156OE vector contains a 35S promoter and the DlamiR156 precursor sequence. Both constructed vectors were introduced into the EHA105 *Agrobacterium* strain, and used for bamboo transformation as we described previously [26]. Briefly, healthy callus was co-infected with *Agrobacterium* for 3 days. Following co-cultivation, the callus tissue was washed with sterile water containing antibiotics. Subsequently, the callus tissue was transferred to a screening medium containing hygromycin and underwent a 3-month resistance screening process to identify positive callus. The positively transformed callus tissue was then used for shoot and root induction. The regenerated plants were verified by qPCR.

To test the impact of miR156 on bamboo shoot organogenesis, calluses with different backgrounds were obtained during the rooting step, and were subsequently cultivated on callus multiplication medium as we described previously [26]. Initial WT callus samples with status similar to that of their counterpart STTM lines and miR56OE lines were rigorously selected, and used for further shoot induction experiments. The regenerated shoots were observed and statistically analyzed after 10 and 20 days of growth on shoot induction medium. At least three independent biological repeats were performed.

### RNA library construction and sequencing

Total RNA from both WT and STTM156 transgenic lines was extracted using TRIzol™ reagent (Thermo, 15-596-026), and its quality, including purity, concentration, and integrity, was thoroughly assessed. The extracted total RNA was utilized for constructing transcriptome libraries. The steps involved in transcriptome library construction were as follows: total RNA extraction, enrichment, and fragmentation of mRNA, cDNA synthesis, end repair, adaptor ligation, and PCR enrichment. Following library construction, the raw sequencing data were obtained, and the adaptor sequences were removed. Quality control of the data was performed using FastQC software. For accurate alignment, high-quality sequencing data were compared with the Ma bamboo genome data using HISAT2 software to obtain the positional information on the reads in the reference genome. Subsequently, HTseq-count software was employed for quantitative analysis of the data. Genes exhibiting |log_2_FC| > .5 and *P*-value <.05 were considered differentially expressed. To gain insights into the functional enrichment of the differentially expressed genes, GO enrichment analysis was performed using the R package DESeq [50].

## Supplementary Material

Web_Material_uhad223
