# Supplementary material for: Uncovering the miRNA-mediated regulatory network involved in Ma bamboo (Dendrocalamus latiflorus) de novo shoot organogenesis
Source: Hortic Res. 2023 Nov 8;10(12):uhad223. doi: 10.1093/hr/uhad223 (PMC10716635; doi:10.1093/hr/uhad223)
Supplement: Web_Material_uhad223 [file web_material_uhad223.zip › SI.pdf]

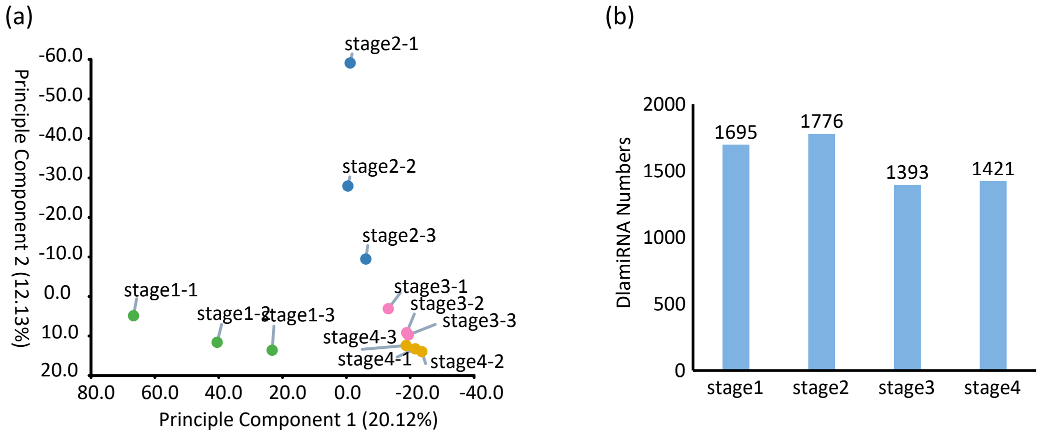

Figure S1 Information of sRNAome. (a) PCA analysis of 12 small RNA libraries. The PCA analysis was conducted on 12 small RNA libraries. The samples were color-coded as follows: Green: stage 1, Blue: stage 2, Pink: stage 3, Orange: stage 4. (b) Number of mature DlamiRNAs identified from the 4 stages of Ma bamboo shoot organogenesis.

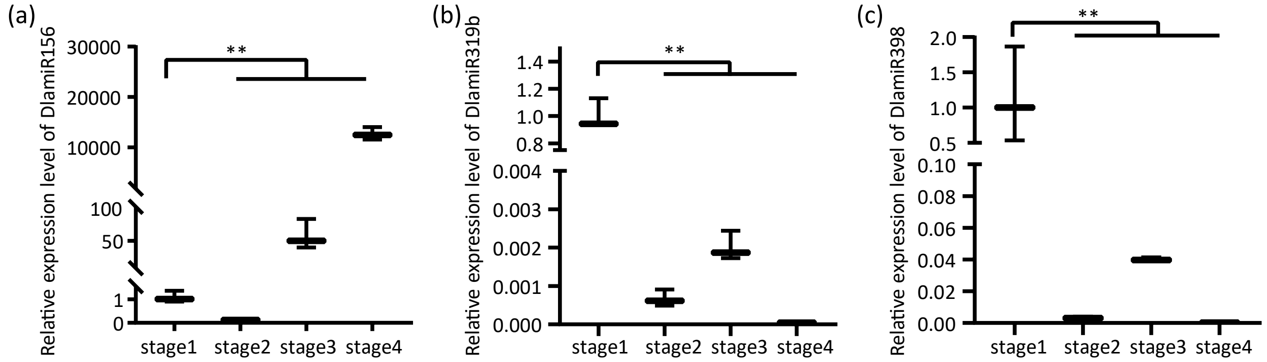

Figure S2 Expression analysis of 3 represented DEM during Ma bamboo shoot organogenesis. Stem-loop RT-qPCR of DlamiR156 (a), DlamiR319b (b) and DlamiR398 (c) during Ma bamboo shoot organogenesis. The expression level of DlamiRNA at stage 1 was assigned a value of 1. Data represents the average of three replicates ± SE. (**, $P < 0.01$).

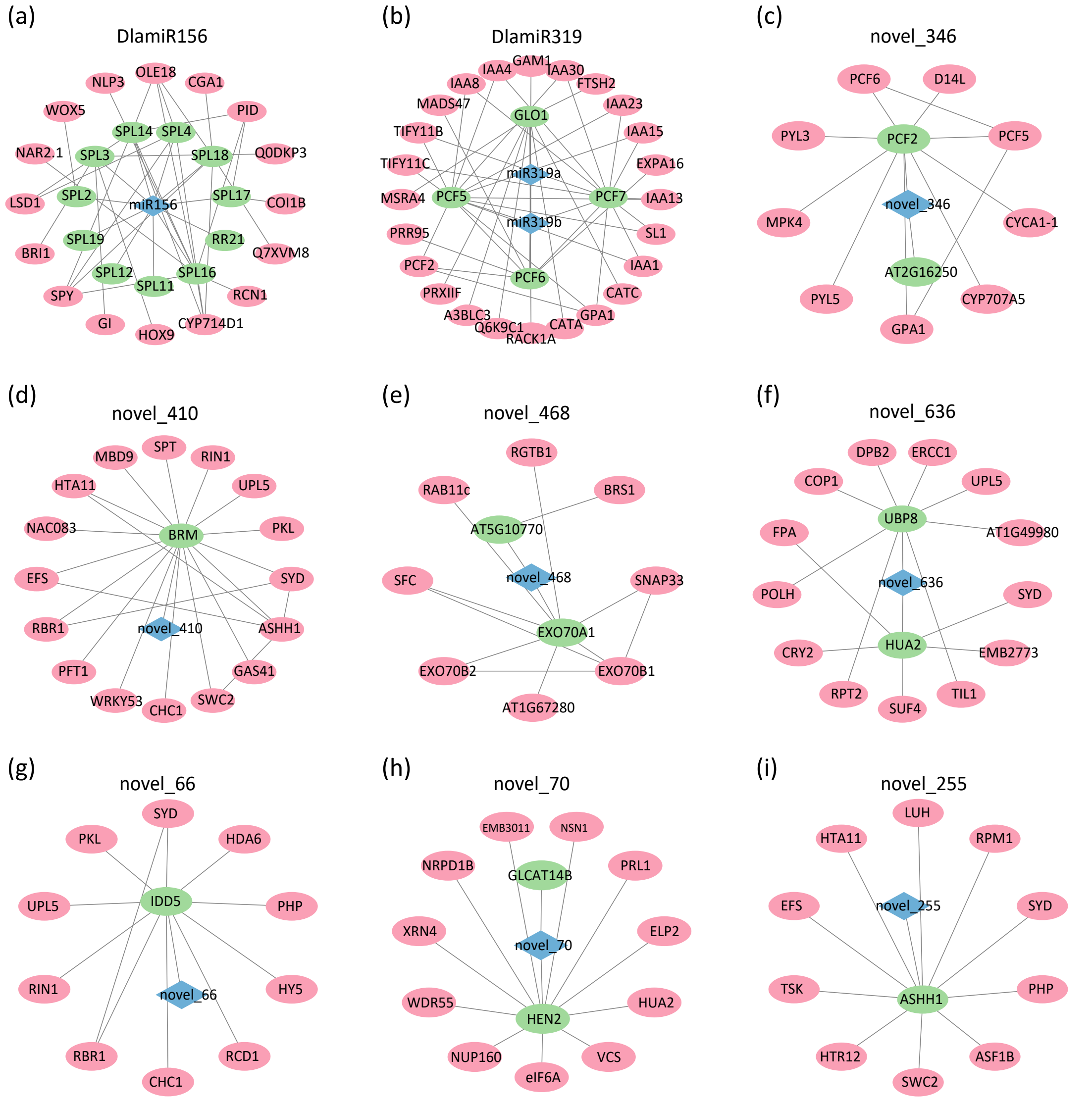

Figure S3 The subnetwork of selected 9 DEM during shoot organogenesis. The subnetwork of DlamiR156 (a), DlamiR319 (b), novel_346 (c), novel_410 (d), novel_468 (e), novel_636 (f), novel_66 (g), novel_70 (h) and novel_255 (i). Blue circles represent DEM during shoot organogenesis, green circles represent DEM target genes and pink circles represent downstream genes.

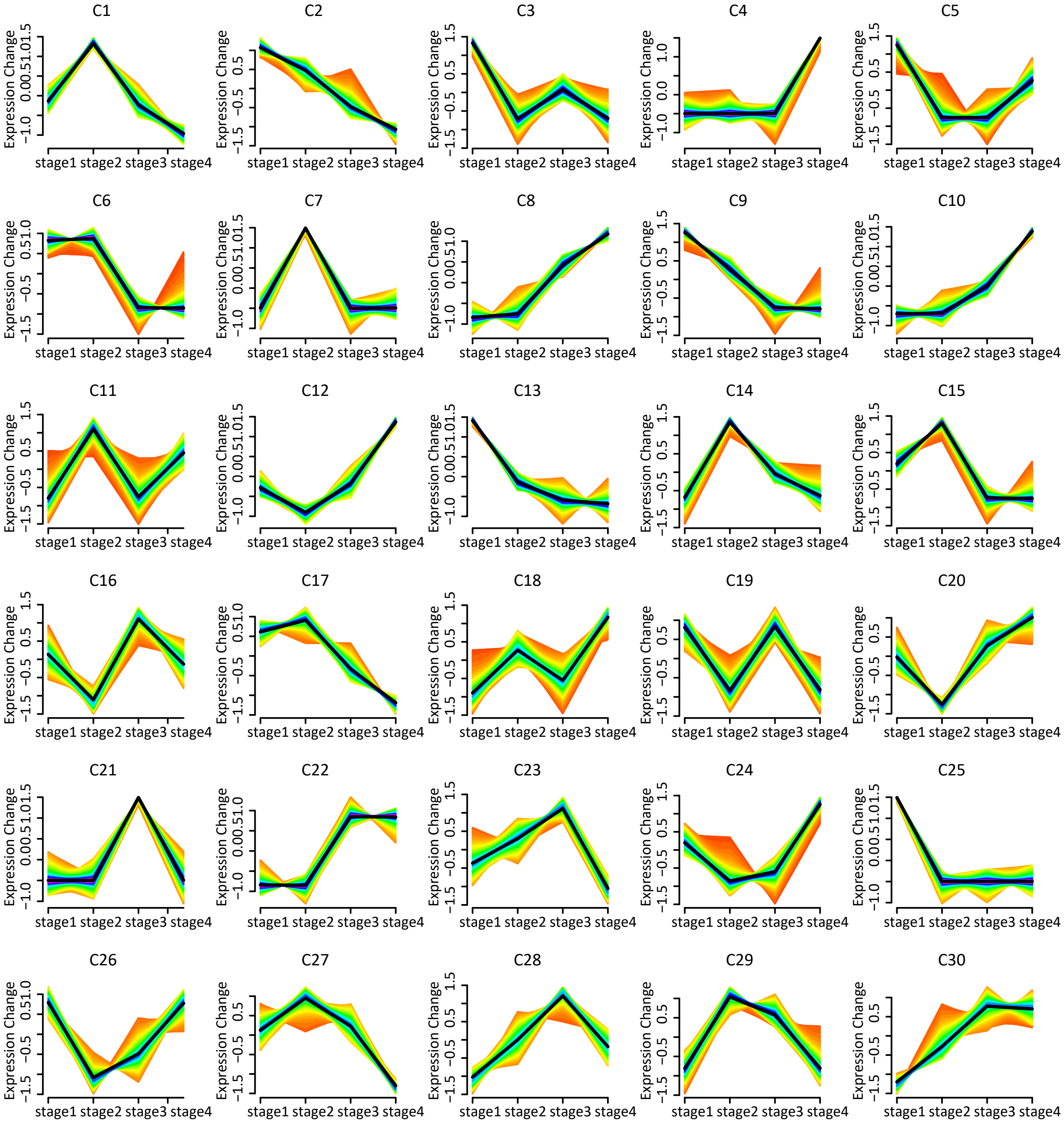

Figure S4 K-means analysis of DEG during shoot organogenesis. The transcriptome data was subjected to K-means analysis, resulting in the division of DEGs into 30 clusters (C1-30) based on their distinct expression patterns.

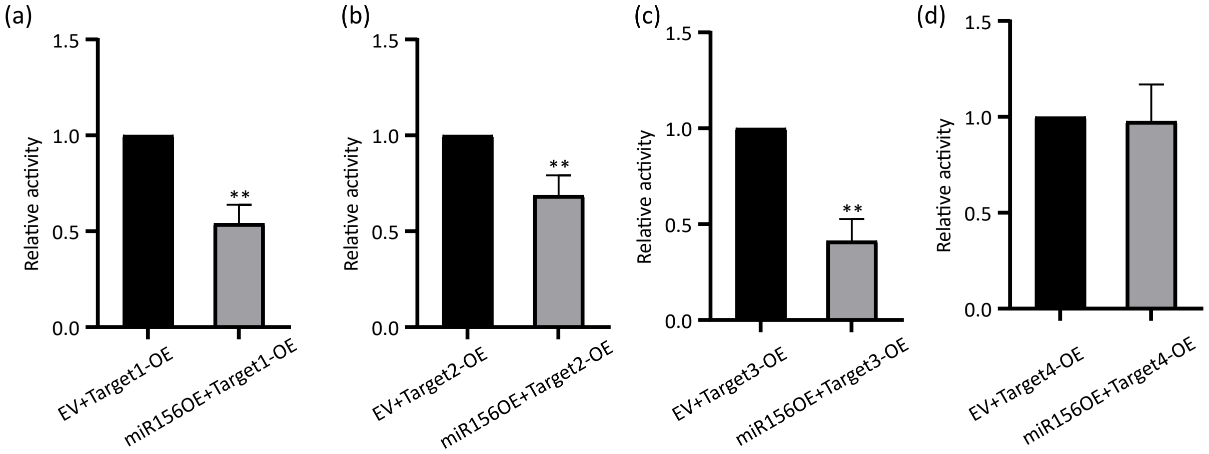

Figure S5 Measurements of the luciferase activity of DlamiR156 and its different site. (a-d) The expression level of EV+Target-OE was assigned a value of 1, and the luciferase activities for target 1 to target 3 (a-c) were measured. Bars represent the mean values of three replicates ± SE. (**, $P < 0.01$).

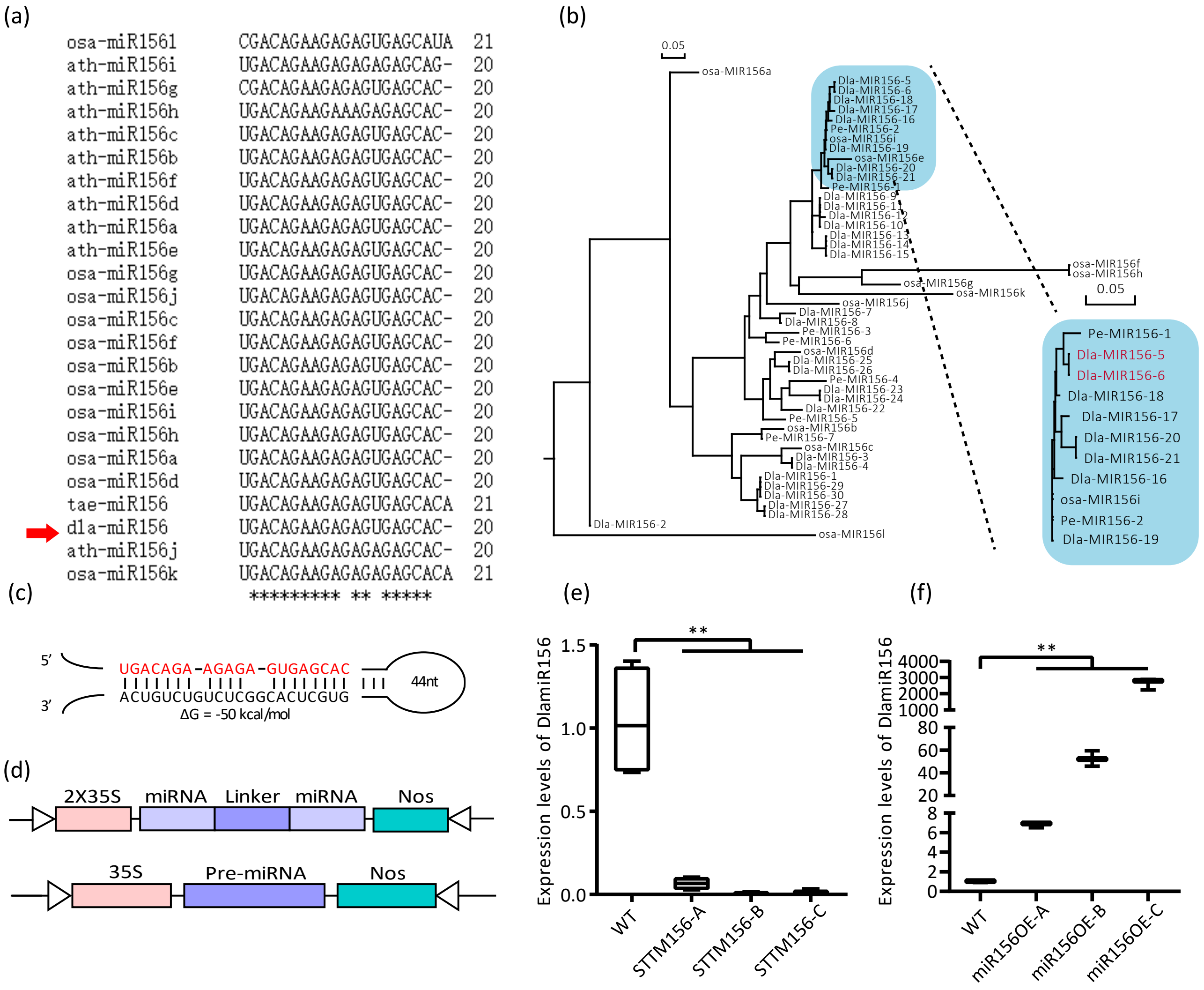

Figure S6 Identification of DlamiR156 from Ma bamboo and generation transgenic bamboos with altered DlamiR156 expression levels. (a) Alignment of mature DlamiR156 sequences of Arabidopsis, rice, maize and Ma bamboo. (b) MIR156 phylogenetic tree derived from rice, Ma bamboo and Moso bamboo (*Phyllostachys edulis*). The phylogenetic tree was generated by the Jukes-Cantor distance method and displayed using DNAMAN software. (c) The secondary stem-loop structure of DlamiR156 precursors in Ma bamboo. Red regions represent mature DlamiR156 sequence. The minimum free energy of the RNA secondary structure was calculated as ΔG (kcal/mol). (d) Diagrams showing STTM156 and miR156OE vectors. (e) Expressions of DlamiR156 in WT and STTM156 transgenic bamboo by stem-loop RT-PCR analysis. The expression level of DlamiR156 in WT was assigned a value of 1. Data represents the average of three independent experiments ± SE. (**, $P < 0.01$). (f) Expressions of DlamiR156 in WT and transgenic bamboo overexpressing DlamiR156 by stem-loop RT-PCR analysis. The expression level of DlamiR156 in WT was assigned a value of 1. Data represents the average of three independent experiments ± SE. (**, $P < 0.01$).

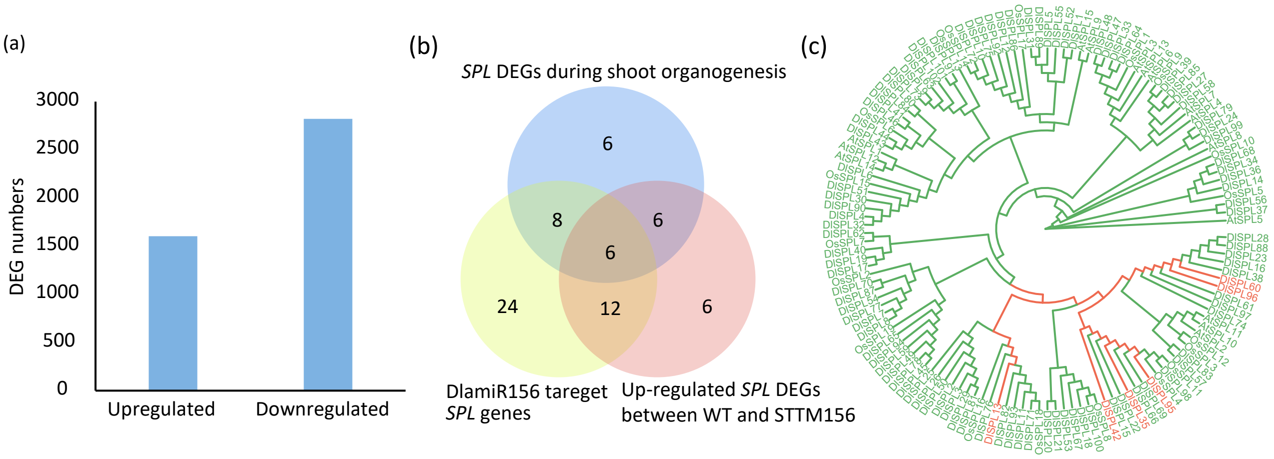

Figure S7 RNA-seq analysis between WT and STTM156 transgenic lines. (a) DEG numbers of upregulated and downregulated genes between WT and STTM156 transgenic lines. (b) Identification of the putative DlamiR156 target *DlaSPL* genes by Venn diagram analysis of the *DlaSPL* genes from the dataset of degradome, transcriptome during shoot organogenesis and transcriptome between WT and STTM156. (c) Distributions of the selected 6 *DlaSPL* genes in the phylogeny tree derived from Arabidopsis, rice and Ma bamboo. Red represents 6 *DlaSPL* genes. Phylogenetic trees was generated by the neighbor-joining method and displayed using MEGA 7.0 software.

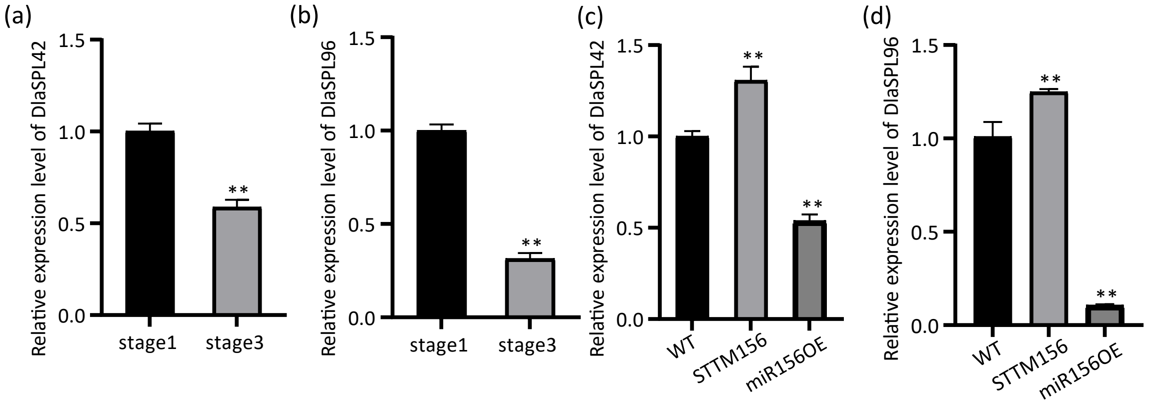

Figure S8 Expressions of 2 putative DlamiR156 target genes. (a-c) qPCR analysis of 2 representative genes (*DlaSPL42*, *DlaSPL96*) in shoot organogenesis of stage1 and stage3. (d-f) qPCR analysis of *DlaSPL42* and *DlaSPL96* representative genes in WT, STTM156 and miR156OE transgenic lines. The expression level of stage1 (a-c) and WT (d-f) was assigned a value of 1. Data represents the average of three independent experiments ± SE. (**, $P < 0.01$).
